# Hydrolytic denitrification and decynidation of acrylonitrile in wastewater with *Arthrobacter nitroguajacolicus* ZJUTB06-99

**DOI:** 10.1186/s13568-018-0719-8

**Published:** 2018-12-03

**Authors:** Yaping Guo, Hui Chang, Qiaoling Wang, Chenjia Shao, Jianmiao Xu

**Affiliations:** 1 College of Environment, Zhejiang University of Technology, Hangzhou, 310014 People’s Republic of China; 2 College of Biotechnology and Bioengineering, Zhejiang University of Technology, Hangzhou, 310014 People’s Republic of China

**Keywords:** Acrylonitrile, Hydrolytic, Denitrification, Decyanidation, Biotreatment, Wastewater

## Abstract

Acrylonitrile (C_3_H_3_N) widely used in chemical raw materials has biological toxicity with –CN bond, so it is the key to removal of cyanide from acrylonitrile wastewater. In our previous research and investigation, a strain was identified as *Arthrobacter nitroguajacolicus* named ZJUTB06-99 and was proved to be capable of degrading acrylonitrile. In this paper, the strain ZJUTB06-99 was domesticated with acrylonitrile-containing medium and its decyanidation and denitrification in simulated acrylonitrile wastewater were studied. The intermediate product of acrylonitrile in degradation process was identified through gas chromatography–mass spectrometer, as well as the biodegradation pathway of acrylonitrile in wastewater was deduced tentatively. The kinetics equation of biodegradation of acrylonitrile was lnC = − 0.1784t + 5.3349, with the degradation half-life of acrylonitrile in wastewater by 3.885 h. The results of this study showed that the optimum levels of temperature, pH and bacteria concentration to attain the maximum biodegradation were obtained as 30 °C, 6 and 100 g/L, respectively. The disadvantages of the biodegradation with this strain and its possible enhanced method to degrade acrylonitrile in wastewater were also discussed.

## Introduction

Acrylonitrile is a kind of highly poisonous organic compound bearing a –CN group that is extensively used in synthesis of rubbers, acrylic fibers, plastics and other synthetic materials (Baxter et al. 2006; Ramteke et al. 2013). Besides, acrylonitrile is third of the 129 priority pollutants listed by EPA (Keith and Telliard 1979; Kumar et al. 2008). The global production of acrylonitrile exceeds 5 million tons, with a proportion of 28% produced in China (Dong et al. 2017; Zheng et al. 2015). Thus, a large amount of acrylonitrile wastewater is generated during the production (Lai et al. 2012), which has inevitably caused serious impact on public and environmental health (Ramteke et al. 2013). Furthermore, these emerging pollution sources are highly toxic, carcinogenic (Ramakrishna et al. 1989; Thier et al. 2000; Quast 2002) and related toxicity investigation has been widely reported, e.g. acrylonitrile could leads to neurobehavioral disorder in rats and some human sicknesses (Caito et al. 2013; Khan et al. 2009; Tanii and Hashimoto 1984). So it is urgent to explore safe and efficient treatment technology on acrylonitrile wastewater treatment.

In the past few years, there are a large number of treatment methods have been developed to degrade acrylonitrile wastewater, including Fenton oxidation (Popuri et al. 2011), supercritical water oxidation (Shin et al. 2009), radiation (Abdel-Aal et al. 2006) and wet air oxidation (Mishra et al. 1995). At present, the biodegradation of acrylonitrile in wastewater is considered to be one of the major routes due to its simple management and high efficiency (Wyatt and Knowles 1995; Kubsad et al. 2011; Santoshkumar et al. 2011). For example, an acrylonitrile-degrading bacterial strain was isolated from the activated sludge of petrochemical wastewater treatment system to investigate its efficiency in acrylonitrile removal (Shakerkhatibi et al. 2010). A novel nitrilase-producing of *Streptomyces* sp. MTCC 7546, was immobilized in agar powder to discuss these cells on biotransformation of acrylonitrile, and the results indicated that the bacteria could efficiently be used for the bioconversion of acrylonitrile to acids without producing amides (Agarwal and Nigam 2017; Nigam et al. 2009). *Rhodococcus rhodochrous* BX2 was possessing the expression of corresponding metabolic enzymes to degrade acrylonitrile completely (Santoshkumar et al. 2011). A newly strain named *Rhodococcus ruber* AKSH-84 was screened from a petroleum-contaminated sludge sample and it has proved to be able to be used in green biosynthesis of acrylic acid for biotechnological processes (Kamal et al. 2011). In a previous research, *Arthrobacter nitroguajacolicus* ZJUTB06-99, capable of converting acrylonitrile to acrylic acid, was isolated in our laboratory (Shen et al. 2009a). Moreover, it was focused on study the biotransformation of acrylonitrile into acrylic acid by using nitrile-converting enzymes as biocatalysts and investigated the optimal medium components of nitrilase production (Shen et al. 2010;  Shen et al. 2009b).

In this work, high concentration of the strain of ZJUTB06-99 was induced in the simulated acrylonitrile wastewater and was centrifuged. Then, it was used in simulated acrylonitrile wastewater to investigate its possibility of decyanidation and denitrification. The possible biodegradation pathway of acrylonitrile and kinetics equation were discussed, separately. Furthermore, the biodegradation effects of acrylonitrile wastewater and their influencing factors were also exhibited. Finally, it was revealed the disadvantages of biodegradation with this strain and discovered the possible enhanced method to degrade acrylonitrile in wastewater, which was contributed to further exploration of practical application in acrylonitrile wastewater.

## Materials and methods

### Materials and medium

Acrylonitrile used in this study was obtained from Aladdin Industrial Inc. (Shanghai, China). All other chemicals were of analytical grade. Glassware was meticulously cleaned to reduce any background contamination.

LB medium for enrichment with the following composition was used: 1.0 L deionized water, 10.0 g glucose, 5.0 g yeast extract, 0.5 g KH_2_PO_4_, 0.5 g K_2_HPO_4_, 0.5 g MgSO_4_·7H_2_O.

The solid medium of LB for isolation contained 1.0 L deionized water, 10.0 g peptone, 5.0 g beef paste, 5.0 g NaCl, 20 g agar.

The pH values of all media were adjusted to 7.2 ± 0.2 with NaOH (1 mol/L), and media were sterilized by autoclaving at 121 °C for 20 min.

### Bacteria growth

ZJUTB06-99 has been deposited in the China Center for Type Culture Collection under accession number CCTCCM208252.

**Fig. 1:**
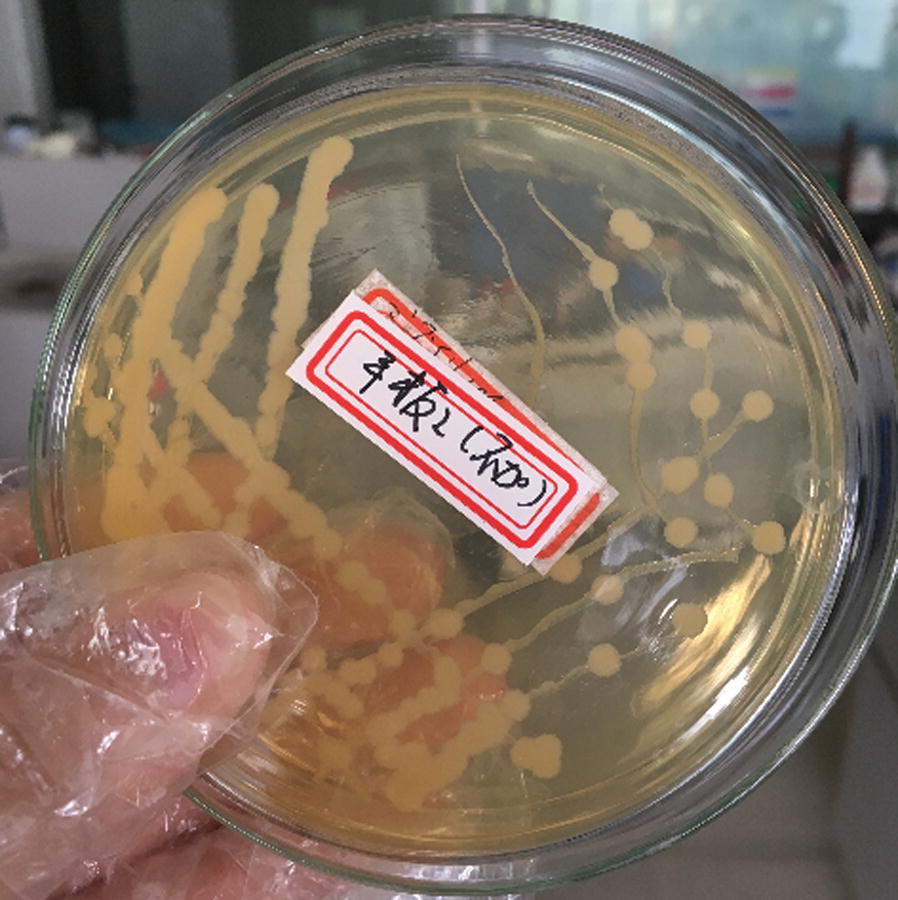
The colony of ZJUTB06-99 on plate

The media used for isolation of *Arthrobacter nitroguajacolicus* from contaminated soil, preservation of the isolated acrylonitrile-degrading strains, was prepared as follows. In this research approach, this strain was cultured on plate medium containing acrylonitrile (200 mg/L) prepared as mentioned above. Approximately 3% of the inocula of the seed medium were inoculated into 250 mL flasks with 50 mL of culture medium and then cultured at 30 °C for 24 h. The strain were harvested by centrifugation (10,000 r/min, 10 min) and washed with deionized water for three times, which was maintained in 15% glycerol solution at − 80 °C for further use. As shown in Fig. 1, the pure colony of ZJUTB06-99 was characterized by a round shape, light milky yellow, neat and smooth edge.

### Optimal experiments

To determine optimal conditions for degrading acrylonitrile by the strain ZJUTB06-99, single-factor optimization tests were designed in this study under different growth conditions including pH (5, 6, 7, 8, 9), temperature (25, 30, 35, 40 °C), bacteria concentration (20, 30, 40, 50, 75, 100 mg/L). The initial acrylonitrile concentration was maintained at 200 mg/L. The residual concentration of acrylonitrile was determined by GC.

### Analytical methods

Reaction sample was collected directly from the reactor with a syringe and passed through a 0.22 μm membrane filter to remove the bacteria. The concentration of acrylonitrile was determined by gas chromatograph (Agilent 6890N, USA) equipped with an automatic injector and a flame ionization detector (FID). The experimental conditions were as follows: the injection temperature was set at 160 °C; and the column and detector temperatures were set at 170 °C and 200 °C, respectively. The intermediate product in the degradation process was analyzed by GC–MS (Agilent 7890/5975, USA), equipped with a HP-5MS capillary column. The GC column was operated in temperature programmed mode at 40 °C for 1 min, raised to 80 °C at a rate of 10 °C/min, and then raised at 210 °C (held for 3 min).

The concentrations of nitrite, nitrate and total nitrogen, ammonia in water samples were assayed according to Chinese Standard Methods. The pH value of the solution was determined with a pH meter. The content of dissolved oxygen was detected by micro-breathing electrode (Unisense, Denmark).

### Reactor system

**Fig. 2:**
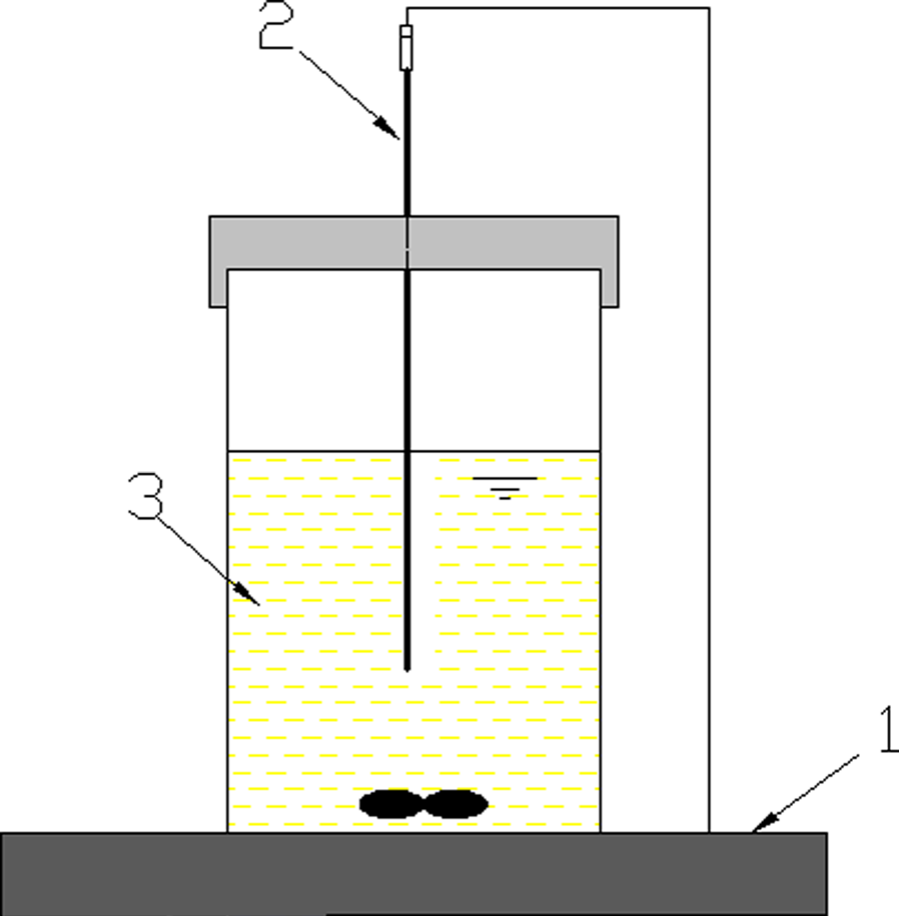
Experimental apparatus: (1) magnetic stirrer; (2) temperature probe; (3) bacteria suspension

The reactor was made of transparent synthetic glass container of 40 mm in length and 40 mm in width and 150 mm in height. Simulative acrylonitrile wastewater with the volume of 200 mL and ZJUTB06-99 were mixed together in a glass container, then put the reactor on a digital thermostat magnetic stirrer with temperature probe. The structure of the reaction apparatus was shown in Fig. 2.

## Results

### Biodegradation pathway of acrylonitrile

In order to understand the biodegradation process of acrylonitrile, the intermediate product during the experiment were tentatively detected by GC–MS. Total ion chromatogram and mass spectrograms were exhibited in Figs. 7, 8, and 9, respectively.

**Fig. 3:**
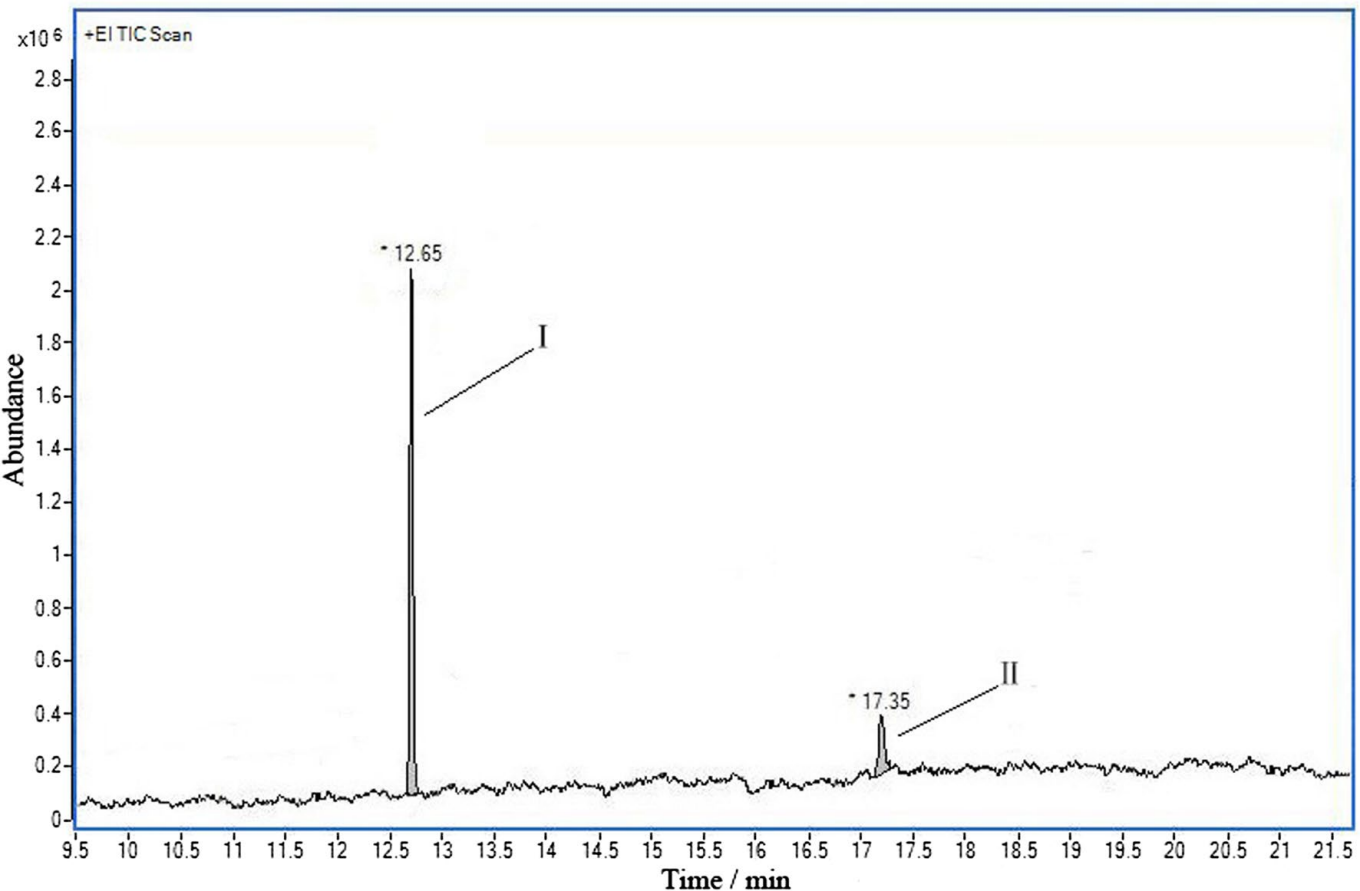
Total ion current chromatogram

There were two peaks with relatively larger response values marked as Peak I and Peak II, as shown in Fig. 3, whose retention time were 12.65 min and 17.35 min respectively.

**Fig. 4:**
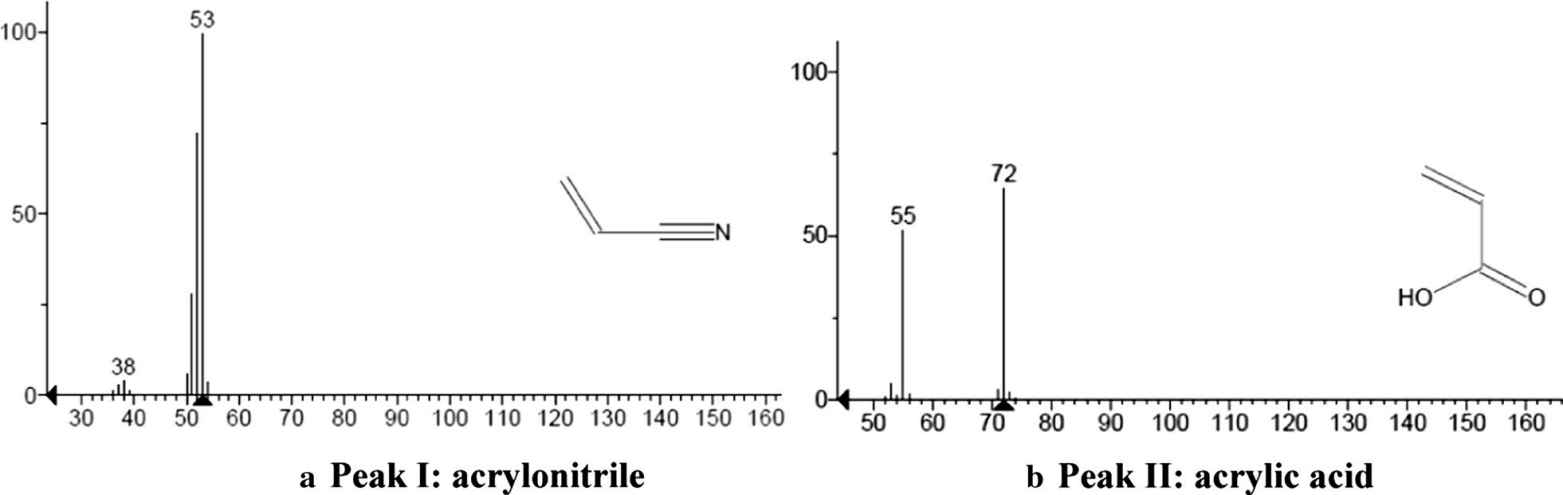
Mass spectrogram of Peak I and Peak II

After searching in the reference mass spectral library, the highest matched mass spectrogram was retrieved according to the peaks in total ion current chromatogram. Peak I was acrylonitrile shown as Fig. 4a and Peak II was acrylic acid shown as Fig. 4b. It can be known that acrylic acid was the main intermediate product during the course of biodegradation.

The concentration of acrylonitrile and acrylic acid in the water samples were detected by GC. The degradation amount of acrylonitrile and production amount of acrylic acid were shown in Table 1.

**Table 1:** The degradation amount of acrylonitrile and the production amount of acrylic acid

Reaction time/(h)	The degradation amount of acrylonitrile/(mmol)	The production amount of acrylic acid/(mmol)
8	0.6163	0.5939

ZJUTB06-99 could degrade acrylonitrile to acrylic acid and the molar ratio of acrylonitrile’s degradation to acrylic acid’s yield was close to 1:1 (Table 1). Besides, it was detected that the content of total nitrogen in the solution was reduced slightly, which was indicated that the ZJUTB06-99 has favorable impact on the removal of –CN bond so as to reduce the toxicity of acrylonitrile. Combining the above results and the previous research on biotransformation reaction of acrylonitrile, it was speculated that the biodegradation process of acrylonitrile wastewater by ZJUTB06-99 conforms to hydrolysis reaction shown as follows:

$$\begin{aligned} \begin{array}{*{20}c} {\text{O}} \\ {||} \\ \end{array} \\ {\text{CH}}_{2} = {\text{CH}} - {\text{C}} \equiv {\text{N}} + {\text{H}}_{2} {\text{O}}{\xrightarrow{{{\text{ZJUTB06-99}}}}}{\text{CH}}_{2} = {\text{CH}} - & {\text{C}} - {\text{OH}} + {\text{NH}}_{3} \\ \end{aligned}$$

### Degradation kinetics

**Fig. 5:**
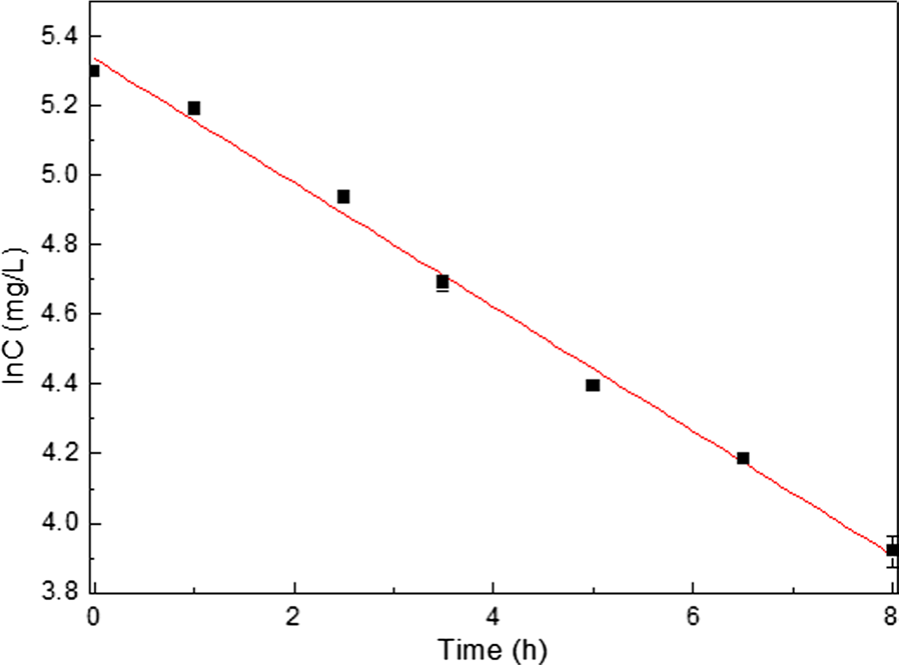
Kinetic curve of degradation

In order to degrade acrylonitrile wastewater with the concentration of 200 mg/L, the experiment was carried out for 8 h and the following degradation curve was shown in Fig. 5. Here, the Y-axis represented to the natural logarithm of acrylonitrile concentration (lnC) and the X-axis represented to reaction time (t). The kinetics equation was lnC = − 0.1784t + 5.3349 with the coefficient of determination R^2^ was determined to be 0.994, suggesting that the experimental data was correlated well with the first order reaction kinetics model. Besides, the k (slope of line) and half-life (t_1/2_) of ZJUTB06-99 was 0.1784 h^−1^ and 3.885 h, respectively.

### Influence of temperature on acrylonitrile biodegradation

**Fig. 6:**
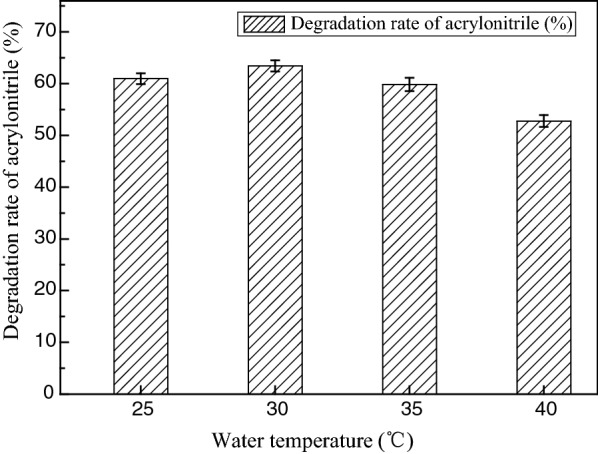
Influence of temperature on acrylonitrile biodegradation rate. The degradation rate increased with the increase of temperature between 25 and 30 °C. A high rate was achieved for ZJUTB06-99 when the temperature was 30 °C

Four different temperatures (25, 30, 35, 40 °C) were thus used to examine the effect of temperature on the biodegradation of acrylonitrile by ZJUTB06-99. As indicated in Fig. 6, the degradation rate increased with the increasing of temperature between 25 and 30 °C. This effect was very pronounced when temperature was varied from 25 to 40 °C. Too high or too low temperature was unfavorable for acrylonitrile degradation.

### Influence of pH on acrylonitrile biodegradation

**Fig. 7:**
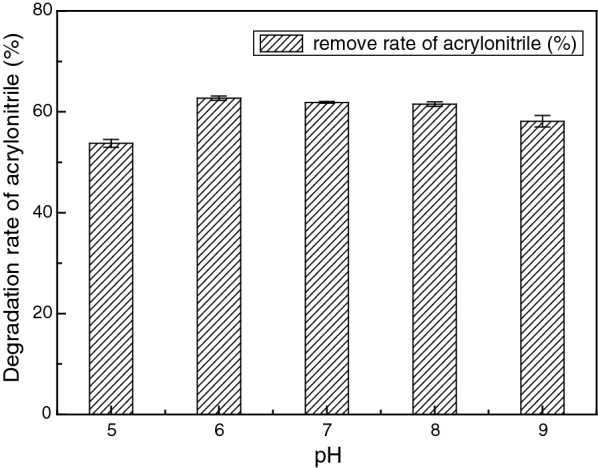
Influence of pH on acrylonitrile biodegradation rate. The rate of acrylonitrile degradation increased when the pH value was increased from 5 to 6. The optimal pH values for ZJUTB06-99 to degrade acrylonitrile were from 6 to 8

The effect of pH ranging from 5 to 9 on the degradation of acrylonitrile was shown in Fig. 7. It was implied that the rate of acrylonitrile degradation increased when the pH value was increased from 5 to 6. The optimal pH values for ZJUTB06-99 to degrade acrylonitrile was 6. The degradation rates from 6 to 8 were higher probably because some key enzymes responsible for acrylonitrile degradation possessed good tolerance at this range of pH.

### Influence of reaction time

**Fig. 8:**
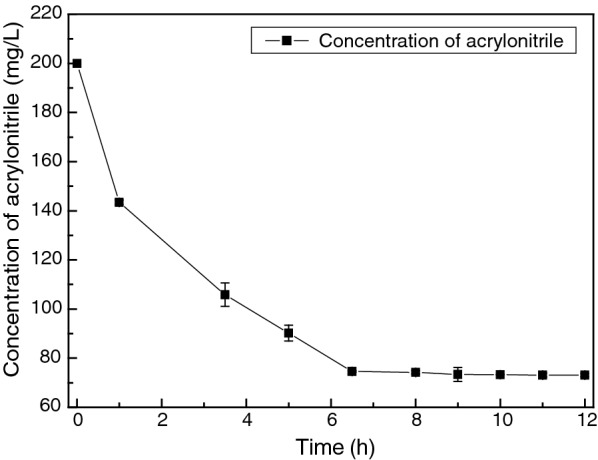
Influence of reaction time on acrylonitrile biodegradation rate. The concentration of acrylonitrile was constantly decreasing with the increase of time. After 8 h, the residual of acrylonitrile decreased slowly and gradually became stable. The optimum biodegradation time was determined to be 8 h

It has been carried out for 12 h during the biodegradation process to investigate the optimal reaction time. As it could be seen from the results in Fig. 8, the concentration of acrylonitrile was constantly decreasing with the increasing of time. After 8 h, the residual of acrylonitrile decreased slowly and gradually became stable. So the optimum biodegradation time was determined to be 8 h.

### Influence of ZJUTB06-99’s concentration

**Fig. 9:**
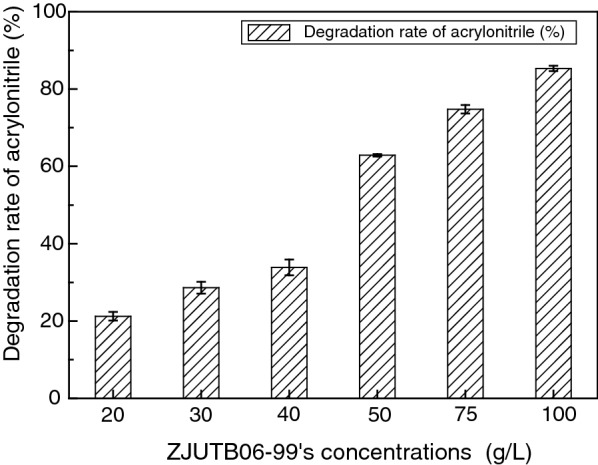
Influence of ZJUTB06-99’s concentration on the biodegradation. The effect of ZJUTB06-99’s concentration on the acrylonitrile’s biodegradation rate. The factor of ZJUTB06-99’s concentration showed the strongest influence on the response when it varied in the range of 20 to 100 mg/L. Especially, the degradation rate of acrylonitrile reached to 85.3% with the ZJUTB06-99’s concentration of 100 g/L. The results could be justified by the higher amounts of effective enzymes that were produced as the concentration of ZJUTB06-99 were increased

The optimization biodegradation on acrylonitrile under different ZJUTB06-99’s concentration was investigated. Figure 9 indicated that the effect of ZJUTB06-99’s concentration on the acrylonitrile’s biodegradation rate. The factor of bacteria concentration showed the strongest influence on the response when it varied in the range of 20–100 mg/L. Especially, the degradation rate of acrylonitrile reached to 85.3% with the ZJUTB06-99’s concentration of 100 g/L. The results could be justified by the higher amounts of effective enzymes that were produced as the concentration of ZJUTB06-99 were increased.

## Discussion

With a growing awareness of the detrimental effects of acrylonitrile on the human and environment health, and its importance in synthetic raw materials, researchers pay great attention to the methods on acrylonitrile biological degradation. It is well known that many microorganisms could use acrylonitrile as carbon or nitrogen sources (Asano et al. 1981) and the main metabolic pathways are converted acrylonitrile to amides by nitrile hydratase (Hughes et al. 1998; Santoshkumar et al. 2017) and to carboxylic acids by nitrilase or by a combination of nitrile hydratase (Bandyopadhyay et al. 1986; Kato et al. 1998).

In this paper, the strain named ZJUTB06-99 was harvested to study its decyanidation and denitrification in simulated acrylonitrile wastewater. This result revealed the investigation of intermediate product and the probably metabolic pathway by ZJUTB06-99 in acrylonitrile wastewater. According to the analysis of GC–MS, the supposed degradation pathway of acrylonitrile by strain ZJUTB06-99 was shown above. The processes could be deduced that acrylonitrile was hydrolyzed to acrylic acid and NH_3_ with the breakdown of –CN bond, indicated that the strain ZJUTB06-99 has better effect on decyanidation to reduce the toxicity of acrylonitrile in wastewater. Figure 5 showed the degradation kinetic equation at acrylonitrile initial concentration of 200 mg/L. Analyzed by Origin 8.0, the degradation kinetics equation in acrylonitrile wastewater by ZJUTB06-99 was lnC = − 0.1784t + 5.3349, and the degradation rate was 0.1784 h^−1^. The result demonstrated that biodegradation reaction fit with the first-order kinetics.

Previous studies revealed that the effect of factors on pollutants degradation by microorganisms were always sensitive to pH and temperature (Wang et al. 2011; Kaira et al. 2015). Environmental pH could determine the biodegradation efficiency by affecting microbial diversity and enzyme’s activity (Antoniou et al. 1990; Lin et al. 2010). For example, The *Bacillus pallidus* strain Dac521 containing nitrilase was induced in the thermophilic bacterium, and the enzymatic activity was found to have a constant between pH 6 and 9, with an optimum lever at pH 7.6 (Almatawah et al. 1999). Temperature influences biological life mainly by affecting the activity of biological enzyme and the mobility of microbial cell membrane (Taniguchi et al. 2017; Huang et al. 2018; Mehak et al. 2016). As the temperature gradually increases, the rate of intracellular enzymatic reactions increases. However, once the temperature exceeds the range that the organism can tolerate, the activity of the organism will decrease and even cause death (Kunze et al. 2014).

In accordance with this, strain ZJUTB06-99 showed high degradation efficiency under a wide range of pH 6–8. Particularly, the degradation rate of acrylonitrile was 62.69% under pH 6. A high rate was achieved for ZJUTB06-99 when the temperature was 30 °C, which was a litter different from the previous study (Shen et al. 2009b). The optimum for temperature, biodegradation time, and concentration of ZJUTB06-99 were obtained at 30 °C, 8 h, and 100 mg/L, respectively.

This work has demonstrated that biology method is cost-effective to treat acrylonitrile wastewater by utilizing ZJUTB06-99, while there still exists some disadvantages in this biodegradation process with strain ZJUTB06-99. It was found that the product of acrylic acid was still not easily biodegradable and the concentration of TN decreased slightly after this degradation. Thus, it is necessary to find a method to degrade acrylonitrile efficiently and decrease the concentration of TN more thoroughly in acrylonitrile wastewater. Based on this work of ZJUTB06-99, our team has studied a coupling technology with electrical catalytic enzyme to enhance the degradation and denitrification of acrylonitrile in wastewater, which hope to be published in this journal later.

## Abbreviations

GC–MS: gas chromatography–mass spectrometer
GC: gas chromatography
EPA: Environmental Protection Agency
